# A Silent Enzootic of an Orthopoxvirus in Ghana, West Africa: Evidence for Multi-Species Involvement in the Absence of Widespread Human Disease

**DOI:** 10.4269/ajtmh.2010.09-0716

**Published:** 2010-04

**Authors:** Mary G. Reynolds, Darin S. Carroll, Victoria A. Olson, Christine Hughes, Jack Galley, Anna Likos, Joel M. Montgomery, Richard Suu-Ire, Mubarak O. Kwasi, J. Jeffrey Root, Zach Braden, Jason Abel, Cody Clemmons, Russell Regnery, Kevin Karem, Inger K. Damon

**Affiliations:** Division of Viral and Rickettsial Diseases, Centers for Disease Control and Prevention, Atlanta, Georgia; Ghana Health Service, Ghana Ministry of Health, Accra, Ghana; Wildlife Division of the Forestry Commission, Accra, Ghana; Virology Department, Noguchi Memorial Institute for Medical Research, College of Health Sciences, University of Ghana, Legon, Ghana; United States Department of Agriculture, National Wildlife Research Center, Fort Collins, Colorado

## Abstract

Human monkeypox has never been reported in Ghana, but rodents captured in forested areas of southern Ghana were the source of the monkeypox virus introduced into the United States in 2003. Subsequent to the outbreak in the United States, 204 animals were collected from two commercial trapping sites in Ghana. Animal tissues were examined for the presence of orthopoxvirus (OPXV) DNA using a real-time polymerase chain reaction, and sera were assayed for antibodies against OPXV. Animals from five genera (*Cricetomys*, *Graphiurus*, *Funiscirus*, and *Heliosciurus*) had antibodies against OPXV, and three genera (*Cricetomys*, *Graphiurus*, and *Xerus*) had evidence of OPXV DNA in tissues. Additionally, 172 persons living near the trapping sites were interviewed regarding risk factors for OPXV exposure, and their sera were analyzed. Fifty-three percent had IgG against OPXV; none had IgM. Our findings suggest that several species of forest-dwelling rodents from Ghana are susceptible to naturally occurring OPXV infection, and that persons living near forests may have low-level or indirect exposure to OPXV-infected animals, possibly resulting in sub-clinical infections.

## Introduction

The first case of human monkeypox (MPX) was identified in 1970 in the Basankusu District of the Democratic Republic of the Congo (former Zaire) during the intensification phase of the smallpox eradication campaign.^1^ In that same year, five additional MPX cases were reported in west Africa, four in Liberia and one in Sierra Leone.^2^,^3^ However, during the subsequent 13 years, only 11 of the 155 MPX cases identified in Africa were reported from west Africa countries (Côte d'Ivoire, Liberia, Nigeria, Sierra Leone);^4^ most cases occurred in the Democratic Republic of the Congo.

The natural cycle of MPX within its native range is poorly understood, as is the route of transmission to humans in the tropical forest setting. The only MPX virus isolated from a wild-caught mammal was obtained from a single moribund rope squirrel (*Funisciurus anerythrus*) collected during an outbreak investigation in Zaire.^5^ Primary human (and non-human primate) infections are hypothesized to result from contact with an infected sylvan animal, although this reservoir host species is currently unknown.

Most of the previous field investigations designed to identify the sylvatic reservoir of MPX virus were confined to relatively short-term outbreak responses and involved general broad spectrum collections of a wide variety of mammalian taxa. The taxa and animals examined were mostly composed of those species that can be purchased from local hunters and collectors. Such taxonomically broad surveys yield large numbers of samples, but usually do not sufficiently sample any individual taxon given the likelihood of a low incidence of active infections in presumptive reservoir species.^6^–^8^

Human MPX has never been described in Ghana, although ecologic niche models predict limited regions of suitable ecology may exist in this country,^9^ and the purported source of the virus introduced into the United States in 2003 was rodents imported from Ghana.^10^,^11^ Fourty-seven confirmed and probable human infections were reported during the outbreak in the United States and at least nine mammal species, representing Old World and New World species, were newly recognized as being capable of hosting infections with monkeypox virus (MPXV).^11^ Members of three genera of African rodent were implicated as the initial, inadvertent, source of importation of the virus into the United States, these being *Cricetomys*, *Graphiurus*, and *Funisciurus* (commonly known as giant pouched rat, African dormouse and rope squirrel, respectively).^11^ Multiple individuals of each of these types of animal were captured in Ghana and sent to the United States for sale as pets. The animals arrived in a single consignment on April 21, 2003. Because the animals had been housed near each another before and during transport to the United States, it was impossible to determine at what point each of the different types of animals might have become infected and which, if any, might have harbored the virus at their time of capture.

To determine the precise geographic origin of the infected animals and to assess whether one or more of the three implicated species might harbor MPXV in nature, we traveled to the area in which the animals were originally trapped and collected additional specimens (from the same time of year), which could then be tested for MPXV. Furthermore, we examined residents of nearby communities for serologic evidence of orthopoxvirus (OPXV) exposure to determine to what extent persons living near these animals might be exposed to the virus. The findings of these investigations are detailed below.

## Methods

### Animal collections.

The origin of the MPXV-infected animals imported into the United States on April 21, 2003, was traced to a single facility in Accra, Ghana. A description of husbandry practices at the facility was undertaken by officials from the Ghanaian Wildlife Division in cooperation with the owner, focusing on animal housing, turnover, shipping, and other practices that could have influenced virus transmission among captive animals.

Two localities outside Accra were identified by the facility owner as being the areas from which the original animals from the April 2003 shipment had been collected. One site (5.98125N, 0.58489E) was near the town of Sogakofe, in the Volta region and the other site (5.78562N, 1.01410W) was near the city of Oda in the Eastern region. In March–April, 2004, animals representing several of the species contained in the 2003 consignment and others commonly found in the two source areas were collected and evaluated for the presence of MPXV and for serologic evidence of prior or current infection.

The collection and processing of animals followed *Methods for Trapping and Sampling Small Mammals for Virologic Testing* published by the Centers for Disease Control and Prevention (CDC). Live animals were handled according to guidelines and recommendations of the CDC Institutional Animal Care and Use Committee and as outlined in The Institutional Animal Care and Use Committee protocol *Field Collections in Zoonoses Investigations*. Small rodents were either trapped live by using live traps (H. B. Sherman Traps, Tallahassee, FL) and locally produced live traps or were captured live by hand. Gross pathologic examinations were followed by a complete necropsy in which blood, heart, liver, kidney, lung, spleen, and skin samples were collected and immediately frozen at −20°C before being transferred to dry ice coolers. Specimens were split, with half remaining at the Noguchi Memorial Institute for Medical Research in Accra and half sent to CDC laboratories in Atlanta.

The CDC laboratories in Atlanta evaluated tissue specimens for evidence of OPXV and MPXV (by using a polymerase chain reaction [PCR] and virus culture) and assayed blood specimens for antibodies (by using an enzyme-linked immunosorbent assay [ELISA]).

### Serosurvey.

Residents living in villages near the two original trapping sites were contacted by Ghanaian Ministry of Health officials and CDC investigators and asked to participate in a research study to evaluate human exposure to OPXV species. Local Ministry of Health workers explained the purpose of the study and read the consent form to prospective enrollees, translating from English when necessary. Parental consent was obtained for persons less than 18 years of age. Adolescents (age = 11–17 years) and children (age = 7–10 years) were asked to provide their assent to participate in the study.

Persons who consented to participate were asked to provide basic demographic information (e.g., age, occupation) and to answer several questions pertaining to illness history, smallpox vaccination status, and exposure to wildlife. In addition, one blood specimen was collected from each study participant. Blood for serologic testing was collected in standard marble-top vacutainer™ tubes and stored refrigerated for not more than 48 hours before being centrifuged. Centrifugation and collection of serum was conducted at the Noguchi Memorial Institute for Medical Research. Serum specimens were divided and frozen; one aliquot was kept at the Ministry of Health laboratories and one was sent to CDC for analysis of OPXV-reactive IgG and IgM.

Persons who participated were compensated for their time and effort. The protocol for this study was reviewed and approved by institution review boards at the CDC in Atlanta (CDC protocol #4043) and at the Noguchi Memorial Institute for Medical Research in Accra.

### Laboratory methods.

Detailed methods for preparation and analysis of tissues using real-time PCR are described elsewhere.^11^ All processing of animal tissues was performed under BioSafety Level 3 conditions. Solid tissues (pooled or individual) were placed in disposable dounce homogenizers (Kendall Large Tissue Grinders; Kendall Company catalog no. 3500SA; Tyco Healthcare, Mansfield, MA). One milliliter of phosphate-buffered saline was added to each measured tissue sample before grinding to enable creation of slurries. Genomic DNA was prepared from an aliquot of each slurry (kit catalog no. 732-6340; Bio-Rad, Hercules, CA). Remaining tissue slurries were stored for future virus isolation (see below).

Nucleic acid samples prepared from pooled tissues were tested by real-time PCR for OPXV DNA before testing individual tissues. The primer/probe sequences were selected from the DNA polymerase gene (E9L; GenBank accession no. L22579) with Primer Express version 1.5 (Applied Biosystems, Foster City, CA). These sequences included OPX forward primer (5′-TCA AAT ATT GAT CGT CCA ACG A-3′), OPX reverse primer (5′-TGG ATG AAT TTC TCA ATA TTA GTT GG-3′), and OPX probe (5′FAM-TAA CAT CCG TCT GGA GAT ATC CCG TTA GA-BHQ1-3′). Primers and probe were synthesized in the Biotechnology Core Facility (CDC) by using standard phosphoramidite chemistry. Each reaction (25 μL) contained 5 μL of template DNA, 0.5 μL of each primer, 0.5 μL probe added to the LightCycler FastStart DNA Master HybProbe (2.5 μL vial 1 [DNA polymerase], 3 μL vial 2 [25 mM MgCl_2_], and 10.25 μL vial 3 [water]; Roche, Indianapolis, IN) and TaqMan Exogenous Internal Positive Control (2.5 μL 10× Internal Positive Control Mix and 0.25 μL 50× Internal Positive Control DNA; Applied Biosystems). Thermal cycling conditions for the ABI7900 apparatus (Applied Biosystems) were one cycle at 95°C for 10 minutes and 45 cycles at 95°C for 15 seconds and 60°C for 1 minute. The PCR amplification is based on fluorescent emission after annealing/elongation (60°C).

Each reaction in the real-time assay was performed in triplicate, and the operator was blinded to the identity of the source DNA. Vaccinia virus Wyeth DNA (50 fg) was used as a positive control and six wells with water were used as negative controls. In all wells, the ABI TaqMan exogenous internal positive control DNA (Applied Biosystems) and primers/probe mixture was included to assay for PCR inhibition that may be caused by the sample. Any tissue pool showing a positive result in the assay was further analyzed at the level of individual tissue (i.e., samples of kidney, eyelid, tongue, brain, blood, heart, liver, gonad, lesion, oral swab, ocular swab, lymph node, feces, skin, and urine). Specimens from individual tissues that had positive results were also analyzed for MPXV-specific DNA signatures (targeting the B6R gene encoding an envelope protein^12^), and an aliquot of the sample slurry was used for virus isolation on cultured BSC-40 cells. Reproducibly positive samples were additionally analyzed for a secondary OPXV DNA sequence (14-kD fusion protein, A27L) by using the Artus Orthopox LC PCR kit (Qiagen, Valencia, CA).^13^ Tissue specimens for which positive results were not reproducible were scored as equivocal; further analysis was not attempted on these specimens. A modified OPXV ELISA was used for animal screening.^11^,^14^

Human serum samples were assessed for IgG and IgM reactive with OPXV antigen in accordance with published methods.^14^ Antibody positivity was measured and compared with reference standards. Any reading that was three standard deviations above the mean of the negative controls was inferred to be positive.

### Statistical analyses.

Epidemiologic and demographic variables and qualitative laboratory findings were analyzed by using parametric and nonparametric statistical tests. Nonparametric statistical tests were used when analyzing subsets of data that were not normally distributed. The Mantel-Haenszel common odds ratio (OR) and Fisher's exact tests (two-tailed) were used for categorical variables, and the Student's *t*-test was used for comparison of means derived from continuous variables. A *P* value < 0.050 was used to measure significance of associations.

## Results

### Investigation of export facility.

**Figure 1.**
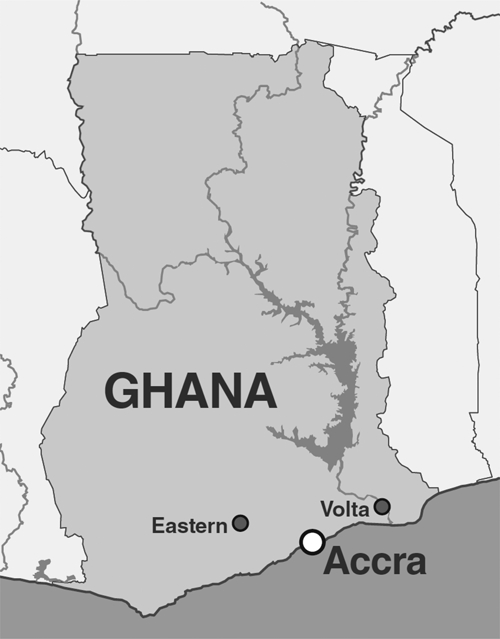
Map of Ghana, showing the two survey locations.

Interviews with the U.S. importer and the licensed commercial Ghanaian exporter indicated that in March–April 2003 collections of multiple Ghanaian mammal species were made by local persons. These animals were subsequently shipped to the United States. The shipment included several rodent species including Gambian rats (*Cricetomys* spp.), rope squirrels (*Funisciurus* spp.), dormice (*Graphiurus* spp.), sun squirrels (*Heliosciurus* spp.), striped mice (*Lemiscomys barbarus*), and brush-tailed porcupines (*Atherurus africanus*). These collections were made gradually over a period of a few weeks. While awaiting shipment, the pouched rats were stored together in concrete enclosures and the squirrels were housed in contiguous wire mesh cages with several individuals per cage. All animals were grouped with members of their own species. Most of the squirrels, and pouched rats were collected in a forest zone northwest of Accra (Figure 1). The potential of a second collection site for at least one of the Genera collected (*Cricetomys*) could not be ruled out by the Ghanaian exporter. The dormice were collected from a lowland scrub vegetation area near the Volta River northeast of Accra. These two sites are separated by approximately 200 miles (Figure 1).

The animal shipments were packed into multi-tiered wooden crates with wire-lined compartments. Members of each of the smaller species (*Lemniscomys* and *Graphiurus*) were shipped with approximately 25 animals per compartment. The exporter reported seeing nothing unusual as far as overt disease in the animals before shipment, nor were any unusual animal die-offs seen at his facility before shipment. The shipment was sent as freight aboard a commercial airline and entered the United States through Dallas, Texas.

### Animals collected during the investigation.

To determine which species of interest might be capable of harboring MPXV in nature, members of the Ghanaian Division of Wildlife Services, CDC, the U.S. Department of Agriculture, and local trappers collected 204 animals over nine nights during March 24–April 2, 2004. Approximately 28% (57 animals) were collected in the Eastern region and the remaining from the Volta region (Table 1). All were apparently healthy adult or sub-adult animals.

Among 182 animals for which serologic analysis was performed, only 2 (one a *Cricetomys* sp. and the other a *Funisciurus* sp.) had detectable levels of antibodies against OPXV at the highest reciprocal serum dilution of 400 (Table 1). One additional *Funisciurus* sp. and one Gambian Sun squirrel (*Heliosciurus gambianus*) had low levels of antibodies against OPXV*.* Moderate levels of antibodies against OPXV were detected in two *Graphiurus* spp. (at a reciprocal dilution of 200), and seven others had low levels of antibodies.

Using the molecular screening algorithm outlined above, we found that 6 of 97 *Graphiurus*, 2 of 38 *Cricetomys*, and 1 of 1 *Xerus* showed evidence of OPXV DNA signatures in harvested tissue specimens (Table 2). An additional 26 other animals, 13 *Graphiurus*, 9 *Cricetomys*, 3 *Funiciurus*, and 1 *Xerus*, showed equivocal results.

Tissues from the nine animals that showed positive results by real-time PCR specific for OPXV (generic) polymerase (E9L) DNA signatures were also assayed for a secondary OPXV DNA target (14-kD fusion protein). Three of the nine animals were also positive for this secondary OPXV (*Xerus* 289, *Graphiurus* 389, and *Graphiurus* 431) (Table 2). All animals were subsequently screened for MPXV-specific signatures by using a real-time PCR designed to detect sequences corresponding to the MPXV envelope protein (B6R). The MPXV-specific DNA signals were not reproducibly detected in any of the specimens. However, the MPXV-specific assay is slightly less sensitive than the OPXV generic A27L assay (10 genomes versus 4 genomes, respectively).^12^,^13^ A cytopathic effect caused by OPXV was not observed in virus cultures prepared from the tissues of any animals, nor was culturable virus obtained after serial passage. (In our hands, virus culture is typically 10–100-fold less sensitive than the most sensitive real-time PCR in detecting OPXV in clinical specimens.)

One *Graphiurus* spp. (animal number 431) had a low level of antibodies against OPXV in its serum and OPXV DNA signatures in its spleen. Two *Funisciurus* spp. with antibodies against OPXV had equivocal OPXV DNA findings in pooled organ specimens in the PCR screening.

### Human serosurvey.

To investigate whether persons working closely with sylvan animals and humans living near animal trapping sites might be at enhanced risk for exposure to OPXVs, we conducted a serosurvey among residents of several villages within walking distance of where the animals implicated in the U.S. outbreak had been originally collected. For purposes of this study, these villages will be referred to as villages A/B (Eastern region) and Village C (Volta region). In all, 65 residents of village C (approximately 92% of the total village population), 100 residents of villages A/B (approximately 33% and 85% of the total village populations, respectively), and 7 persons who were directly involved in the exotic animal trade consented to participate in the serosurvey (Table 3). The sex distribution and occupations of study participants were similar between the Eastern Region and Volta region survey sites, but the proportion of persons greater than 23 years of age enrolled from the Volta region was higher than that from the Eastern region.

Most village residents reported that they entered the forest at least once a week (98% and 95% for villages A/B and C, respectively), whereas five of six of those actively engaged in the animal trade reported only going into the forest on a monthly basis. Cultivation and firewood collection were cited most frequently as the principal reason for participants going into forested areas (n = 76 [71%] and n = 28 [26%], respectively), among those 107 participants who provided a response to the question. A higher proportion of men than women cited cultivation as the reason for entering the forest (67% versus 33%, respectively); this bias was of similar proportion in the Eastern and Volta regions. Other reasons cited for forest entry were animal trapping related activities (n = 2) and accompanying adults (n = 1).

All enrolled study participants provided blood for quantification of IgG and IgM against OPXV. Serologic tests used in this study do not detect specific antibodies against MPXV but do detect antibodies broadly cross-reactive against antigens from multiple OPXVs, including MPXV and vaccinia virus (the live virus component of the smallpox vaccine).

**Figure 2.**
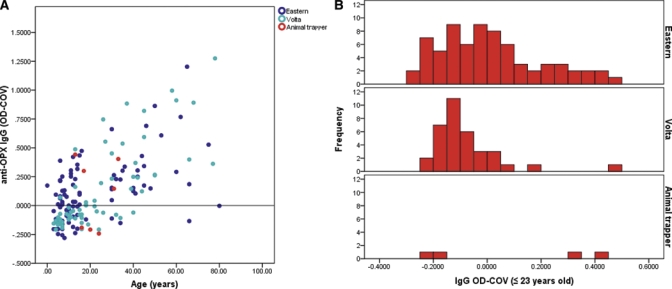
Levels of serum IgG against orthopoxvirus (OPXV) (OD-COV) among study participants in Ghana, by age and region. **A**, Scatterplot depicting OPXV IgG levels by age (years) across the study population. Blue = Easter region; cyan = Volta region; red = persons engaged in exotic animal trade. **B**, Histograms showing the distribution of OD-COV values among persons ≤ 23 years of age, stratified by region, including persons ≤ 23 years of age engaged in exotic animal trade.

None of the study participants had positive results for IgM against OPXV, which suggested that none had been exposed to an OPXV (for which they had been previously immunologically negative) within the prior two months.^14^ However, 91 (53%) study participants had detectable levels of IgG against OPXV (Figure 2A). A higher proportion of those greater than 23 years of age, as opposed to those younger, had detectable IgG against OPXV (n = 51 [81%] versus n = 40 [37%], respectively). This finding is consistent with the fact that many of the older persons in this study would likely have had the opportunity to have received smallpox vaccination (or may even have been exposed to variola virus before smallpox eradication). Smallpox vaccination typically results in life-long persistence of IgG against OPXV. The 24 study participants who reported having received smallpox vaccination at some point in life all had detectable IgG against OPXV and visible vaccination scars; all were greater than 23 years of age.

The proportion of adults (greater than 23 years of age) with positive findings for IgG against OPXV was relatively similar in study participants from the Eastern and Volta regions (87% and 77%, respectively). However, among persons ≤ 23 years of age, there was a significantly greater probability of a positive finding for IgG against OPXV among those living in the Eastern region (OR = 4.05, 95% confidence interval [CI] = 1.50–11.03) (Table 4 and Figure 2B). Those persons engaged in the exotic animal trade were not more likely to have IgG against OPXV than village residents (independent of age). Sex or reported frequency of visits into forested areas were not individually associated with IgG against OPXV.

The principal reason persons cited for going into the forest was associated with status of IgG against OPXV, but in an age-delimited fashion. It has been estimated that half of boys and girls 10–14 years of age in Ghana are substantively engaged in farming and domestic work, which is double the proportion of those 7–9 years of age involved in these activities.^15^ Among study participants greater than nine years of age (persons anticipated to have significant levels of engagement in the activity cited) with no history of smallpox vaccination, cultivation, rather than firewood collection, was found to be significantly associated with IgG against OPXV (OR = 3.24, 95% CI = 1.00–10.47) (Table 4).

**Figure 3.**
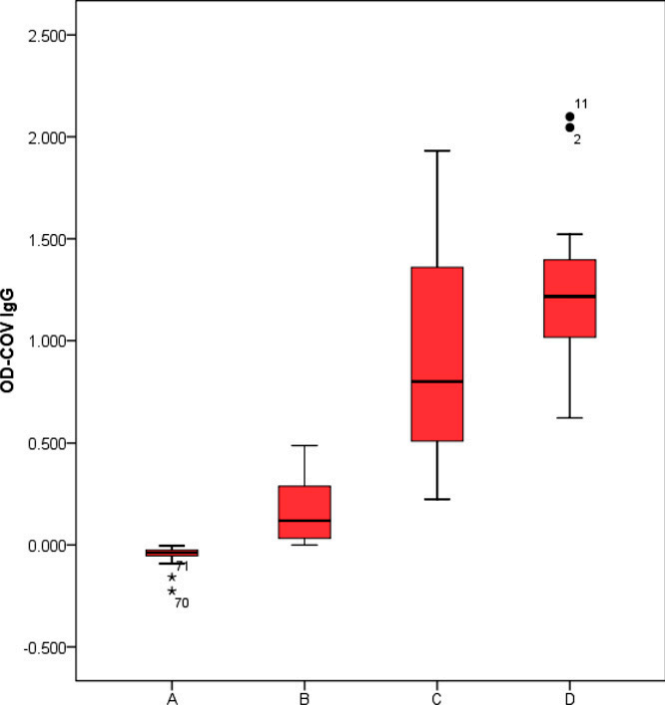
Box plots of levels of serum IgG against orthopoxvirus (OPXV) (OD-COV) for **A**, non–smallpox-vaccinated persons who were not infected with monkeypox virus (MPXV) infection by clinical, epidemiologic, and laboratory criteria during the MPX outbreak in United States in 2003 (n = 74); **B**, persons positive for IgG against OPXV ≤ 23 years of age in this study (n = 40); **C**, persons confirmed as having MPXV infection during the U.S. outbreak approximately one year post-illness onset (n = 14); and **D**, persons confirmed as having MPXV infection during the U.S. outbreak with acute-phase illness (n = 15).^16^ This figure appears in color at www.ajtmh.org.

Evaluation of absolute levels of IgG against OPX in the study population showed that in persons ≤ 23 years of age, levels were substantially below that which would be anticipated in persons with recent acute infection with OPXV (Figure 3). To assess whether the relatively low ELISA optical density values observed in positive for IgG against OPXV across the study population were greater than would be expected from a known OPXV-negative population, the mean OPXV IgG level among IgG-positive persons ≥ 23 years of age from Ghana (the group with the lowest serum level of IgG against OPXV [OD-COV], n = 40) was compared with that of specimens obtained from persons not vaccinated against smallpox in the United States (persons ruled out for MPXV infection in 2003 by clinical, epidemiologic, and laboratory criteria, n = 74).^16^ Although substantially lower than the mean OD-COV for persons acutely infected with MPXV and persons one year post-infection, the mean OD-COV for IgG-positive Ghanaians ≤ 23 years of age (OD-COV = 0.17) was significantly higher than that for OPXV-negative persons in the United States (OD-COV = −0.05; *P* < 0.001 by two-sided Student's *t*-test, degrees of freedom = 41).

Although overall levels of IgG against OPXV among IgG-positive persons in this study were low relative to what is typically observed within one year of acute OPXV infection, six persons in this study showed highly elevated levels of IgG against OPXV (defined here as OD-COV > 2 standard deviations above the mean of the IgG-positive population in the study). All of these persons were greater than 23 years of age and described themselves as farmers. Five were from the Volta region; they reported having received smallpox vaccinations. One was from the Eastern region; this person did not report receiving a smallpox vaccination and had no evidence of a vaccine-associated scar.

## Discussion

Somewhat contrary to expectations, our results did not implicate a single species of animal as being the sylvan source of MPXV for the U.S. outbreak. We found evidence suggestive of intermittent exposure to OPXVs among various species across a broad geographic span. Species of animal showing evidence of OPXV exposure (i.e., *Cricetomys* spp., *Graphiurus* spp., *Funiscuris* spp., *Xerus* spp.) mirrored those implicated in the U.S. outbreak,^11^ with the exception of the *Xerus* spp., which had not previously been associated with OPXV exposure. It should be emphasized that our study was not intended to constitute a comprehensive survey of the sylvan fauna of Ghana for evidence of OPXV infection. Only rodent species were evaluated. Species implicated in the U.S. outbreak were preferentially collected. Many of the species were sampled below the threshold needed to determine whether their OPXV nucleic acid and seroprevalence rates were comparable to those observed for *Graphiurus* spp., the most well sampled species (for which nucleic acid and antibody prevalence rates of 6.2% and 10%, respectively, were observed).

If we assume that rodents, like humans, will sustain life-long detectable OPXV antibody titers after infection, the relatively low seroprevalence rates observed for two of well sampled species of interest, *Graphiurus* spp. and *Cricetomys* spp. (2.6% and 10%, respectively), might indicate that these species are unlikely to serve as natural sylvan reservoirs for OPXVs. Members of the genus *Funisciurus*, which showed a seroprevalence of 40%, might be good candidates. The only isolation of MPXV from an animal in the wild came from a *Furnisciurus anerythrus*,^5^ further indicating the relevance of this species as a putative maintenance source of the virus in nature. However, studies of other, better-characterized, rodent-borne zoonotic viruses have documented similarly low point seroprevalence rates.^17^ Additionally, ecologic investigations of OPXVs from Europe performed in the United Kingdom, which investigated the influence of cowpoxvirus infection on population cycles in the reservoir host (the field vole *Microtus agrestis*), have shown that population seroprevalence rates can vary temporally, thus limiting the informational value of single cross-sectional measurements to implicate, or rule out, virus maintenance hosts.^18^ In one such study,^19^ sharp decreases in seroprevalence were observed in bank vole populations that had experienced recent demographic crashes. The investigators noted that in some instances population seroprevalence decreased to undetectable levels after approximately two-fold decreases in density during the period six months prior, and conversely, reached as high as approximately 90% in the post-rebound period (in delayed density-dependent fashion, with a lag occurring between population rebound and increased seroprevalence). Therefore, understanding the interplay between MPXV and the population dynamics of various candidate reservoir species will be crucial to arriving at an accurate interpretation of OPXV serologic and virologic findings.

Animal infection experiments that illuminate features of virus transmission dynamics and pathobiology in various candidate reservoir species can also be helpful in identifying the reservoir host, and differentiating it from other species that are merely permissive of virus replication. The interpretation of human serologic findings is similarly nuanced. Also, contrary to expectations, the results of our study did not show enhanced risk of OPXV exposure among persons directly involved in the exotic animal trade. Instead, we observed IgG against OPXV among a large proportion of those persons living in rural communities near the original animal trapping sites, albeit no persons had serologic evidence suggesting recent infection. Smallpox vaccination continued in Africa until 1980. Therefore, a substantial proportion of persons greater than 23 years of age at the time that this study was performed would be anticipated to retain cross-reactive antibodies because of vaccination. The presence of antibodies against OPXV in persons born after cessation of vaccination is of somewhat greater interest. In this study, 36% of young non-vaccinated persons had IgG against OPXV. Within this group, mean values for absolute IgG (0.17, 95% CI = 0.12–0.22; derived from a single-dilution ELISA OD values) decreased between those observed for OPXV-negative persons (–0.05, 95% CI = –0.04 to –0.06) and those anticipated for persons one year after having clinically defined MPXV infection (0.80, 95% CI = 0.93–1.25) (Figure 3).

These comparison groups (OPXV-negative persons and persons with clinically defined MPXV infection) are derived from investigations performed during the U.S. MPX outbreak.^14^,^20^ The constituent population for this study is different in many ways from that that found in the United States, but in theory members of both groups, those exposed to MPXV-infected animals in the United States and Ghanaians living near the animal trapping sites, would have had opportunities for exposure to the same strain of MPXV. The question of whether the intermediate levels of seropositivity observed in many young or otherwise unvaccinated persons in this study might be a consequence of asymptomatic or sub-clinical infections with this strain of MPXV is worthy of consideration. Asymptomatic MPXV infections in members of young age groups have been previously hypothesized,^21^ and three persons affected during the U.S. outbreak are presumed to have had sub-clinical MPXV infections.^22^ However, all three of these persons had had prior smallpox vaccination and each had a robust IgG titer against OPXV.

Another possibility to account for intermediate levels of seropositivity is that perhaps beginning at a young age, persons living in the areas surveyed may be exposed to other, as yet uncharacterized, OPXVs that are non-pathogenic in humans. Demonstration of this phenomenon emerged in the 1970s when Baxby and others^23^ demonstrated that young camels, which were fully susceptible to infection with camelpox virus, could not be productively infected with variola virus. However, exposure to variola virus by scarification resulted in production of intermediate-level titers of antibodies against OPXV in these animals,^23^ which were sufficient to provide protection against subsequent challenge with camelpox virus. Taterapox virus is one example of an OPXV that has been identified in west Africa that has an unknown pathogenic capacity in humans.^24^

In general, a broad comparison between the age-specific seroprevalences seen in this study from Ghana versus one performed in an MPXV-endemic setting in the Democratic Republic of the Congo in 1981^21^ showed similar progressive increases in age-specific seroprevalence through successive older age classes, but the overall proportion of seropositive persons at each age class was considerably elevated in this study as opposed to the former study. Similar to the study in the Democratic Republic of the Congo, children in this study from communities surrounded by dense forest (Eastern region) had a significantly higher probability of being seropositive than did children from villages surrounded by mosaic forest-savannah habitat. The habitats in these different areas tend to support varied abundances of several common animal species, including those species of rodent implicated in the MPXV importation event to the United States; *Funisciurus* spp. and *Cricetomys* spp. were more commonly trapped in the Eastern locations and *Graphiurus* sp. was more commonly trapped in the Volta Region. The potential for differing intensities of human exposure to rodent species harboring MPXV exists between the two areas, but has yet to be demonstrated. Cultivating crops in forested areas was also significantly associated with the presence of antibodies against OPXV (in non-vaccinated persons greater than nine years of age). This finding could also be associated with enhanced opportunities for contact with sylvan rodents (crop pests). Alternatively, cultivation activities could merely signal more frequent and longer durations of time spent by persons in a forested habitat.

The lack of an appropriate urban Ghanaian population sample against which to compare human serologic findings and risk factor data limits our ability to investigate absolute risks for OPXV exposure attributable to living in rural communities. Also, the cross-sectional nature of our study precluded our ability to fully explore the possibility that some OPXV-seropositive persons may have had sub-clinical OPXV infections, a question better addressed in a prospective investigation. Other limitations preventing broader interpretation of our study findings include the fact that not all questions were answered by all participants, and that our survey investigation tool was insufficient to capture reliable (non-biased) individual responses to questions about animal exposure.

In summary, the accumulated findings from this study lead us to conclude that no one species of animal and neither collection site could be strongly implicated, nor dismissed, as being the source for the MPXV imported into the United States in 2003. Rather, our results are suggestive of geographically widespread exposure to OPX among rodents found at both sites, near both communities surveyed, leading us to postulate that persons living in these communities may have enduring, but potentially low-intensity or indirect, exposure to OPXV- infected animals resulting in sub-clinical (i.e., asymptomatic) infection concomitant with seroconversion. Possibilities for exposure are likely to begin at a young age, with additional risks present for children living in heavily forested areas. The presence of substantial clinical illness associated with MPXV infection among persons in the United States might be explained by the virus having passed through an amplification host, namely prairie dogs. Contact with MPXV-infected prairie dogs was identified in each instance of human infection, whereas no persons coming into contact with infected African rodents became infected.^22^ We cannot discount the possibility that other uncharacterized OPXVs are also circulating in southern Ghana. This possibility should also be thoroughly explored.

## Figures and Tables

**Table 1:** Results of laboratory testing to detect presence of orthopoxvirus DNA and antibody against orthopoxvirus from tissue and blood specimens of rodents collected in two regions of Ghana*

Genus	No.	Region	Orthopoxvirus DNA detection by real-time PCR†				Orthopoxvirus antibody ELISA endpoint titers				
			Positive‡	Equivocal§	Negative	% Positive	1:50	1:100	1:200	1:400	No. tested
*Graphiurus*	100	Volta	6	13	78	6	7¶	0	2	0	90
*Cricetomys*	40	Eastern	2	9	27	5	0	0	0	1	38
*Lemniscomys*	24	Volta	0	0	24	0	0	0	0	0	20
*Tatera*	9	Volta	0	0	9	0	0	0	0	0	9
*Funiscirus*	9	Eastern	0	3	6	0	1	0	0	1	5
*Mus*	8	Volta	0	0	8	0	0	0	0	0	8
*Heliosciurus*	7	Eastern	0	0	4	0	1	0	0	0	7
*Arvicanthis*	6	Volta	0	1	6	0	0	0	0	0	4
*Xerus*	1	Eastern	1	0	0	100	0	0	0	0	1

* PCR = polymerase chain reaction; ELISA = enzyme-linked immunosorbent assay.

† The orthopoxvirus DNA detection threshold for the orthopoxvirus generic E9L polymerase real-time PCR assay is ≥ 2 fg of purified vaccinia DNA.

‡ Result replicated in independent assay; represents finding from liver, spleen, skin, and/or pooled organ specimens. Details are shown in Table 2.

§ < 3 of 3 replicated positive for pooled organ specimen.

¶ Female dormice were significantly more likely to have Opx-reactive titers at 1:50 than males (*P* = 0.030, by Fisher's exact test, 2-sided).

**Table 2:** Characteristics of animals that had evidence of infection with orthopoxvirus, Ghana*

Genus and animal	Sex	Age	Tissue	E9L PCR	A27L PCR	Orthopoxvirus ELISA titer 1:50
*Graphiurus* 326	M	Adult	Pool	Positive	Negative	Negative
*Graphiurus* 369	M	Adult	Pool	Positive	Negative	Negative
*Graphiurus* 389	F	Adult	Spleen	Positive	Positive	Negative
			Liver	Positive	Negative	Negative
*Graphiurus* 404	M	Adult	Lung	Positive	Negative	Negative
*Graphiurus* 431	F	Adult	Spleen	Positive	Positive	Positive
*Graphiurus* 485	F	Adult	Skin	Positive	ND	Negative
			Pool	Positive	Negative	Negative
*Cricetomys* 459	F	Sub-adult	Spleen	Positive	Negative	Negative
*Cricetomys* 460	F	Sub-adult	Spleen	Positive	Negative	Negative
*Xerus* 289	F	Adult	Spleen	Positive	Positive	Negative

* PCR = polymerase chain reaction; ELISA = enzyme-linked immunosorbent assay; ND = not determined.

**Table 3:** Characteristics of persons from the Eastern and Volta regions of Ghana who participated in the serosurvey

Characteristic	Category No. (%)			Total (%)
	Eastern region resident	Volta region resident	Exotic animal professional	
Total	100 (100)	65 (100)	7 (100)	172 (100)
Sex				
M	54 (54)	37 (57)	6 (86)	97 (56)
F	46 (46)	28 (43)	1 (14)	75 (44)
Age, years*				
≤ 23	70 (70)	35 (54)	4 (57)	109 (64)
> 23	30 (30)	30 (46)	3 (43)	63 (36)
Prior smallpox vaccination†				
Yes	11 (11)	12 (19)	1 (14)	24 (14)
No	89 (89)	52 (81)	6 (86)	147 (86)
Occupation				
Exotic animal trader	–	–	7 (100)	7 (4)
Farmer	30 (30)	30 (46)	–	60 (35)
Commercial	1 (1)	–	–	1 (1)
Child/student	69 (69)	35 (54)	–	104 (60)
Forest visits‡				
At least once a week	95 (97.9)	61 (95.3)	1 (14.3)	157 (93.5)
At least once a month	2 (2.1)	3 (4.7)	6 (85.7)	11 (6.5)

* Routine smallpox vaccinations in Africa ceased in 1982. Persons less than 24 years of age at time of enrollment in this study were not expected to have had the opportunity for childhood vaccination to protect against smallpox.

† Vaccination status is based on self-reporting. Vaccination scars were noted in 22 (92%) of 24 persons who provided a self-report of smallpox vaccination; one person from the Volta region with evidence suggestive of a vaccination scar denied having been vaccinated. This person was not included in either category.

‡ Four persons enrolled in the study did not provide information pertaining to frequency of forest visits.

**Table 4:** Characteristics associated with IgG against orthopoxvirus among study participants*

Characteristic	Orthopoxvirus ELISA		OR (95% CI)
	No. (%) positive	No. (%) negative	
≤ 23 years of age†			
Eastern	32 (84.2)	38 (56.7)	4.07 (1.50–11.03)
Volta	6 (15.8)	29 (43.3)	–
> 23 years of age			
Eastern	26 (53.1)	4 (36.4)	1.98 (0.51–7.63)
Volta	23 (46.9)	7 (63.6)	–
Professional animal worker			
≤ 23 years of age	2 (50)	2 (66.7)	–
> 23 years of age	2 (50)	1 (33.3)	–
Primary reason for forest visits†			
Cultivation	31 (86.1)	23 (65.7)	3.24 (1.00–10.47)
Firewood	5 (13.9)	12 (34.3)	–

* ELISA = enzyme-linked immunosorbent assay; OR = odds ratio; CI = confidence interval.

† Among persons > 9 years of age with no history of smallpox vaccination.
